# An Efficient and Compact Difference-Frequency-Generation Spectrometer and Its Application to ^12^CH_3_D/^12^CH_4_ Isotope Ratio Measurements

**DOI:** 10.3390/s100706612

**Published:** 2010-07-09

**Authors:** Kiyoshi Tsuji, Hiroaki Teshima, Hiroyuki Sasada, Naohiro Yoshida

**Affiliations:** 1 Department of Environmental Science and Technology, Interdisciplinary Graduate School of Science and Engineering, Tokyo Institute of Technology/4259, Nagatsuta-cho, Midori-ku, Yokohama, 226-8502, Japan; E-Mail: yoshida.n.aa@m.titech.ac.jp (N.Y.); 2 SENTAN, Japan Science and Technology Agency/Sanbancho 5, Chiyoda-ku, Tokyo 102-0075, Japan; 3 Department of Physics, Faculty of Science and Technology, Keio University/3-14-1, Hiyoshi, Kohoku-ku, Yokohama, 223-8522, Japan; E-Mails: hiroaki@z3.keio.jp (H.T.); sasada@phys.keio.ac.jp (H.S.)

**Keywords:** isotope ratio, methane, laser spectroscopy, difference frequency generation, waveguide PPLN

## Abstract

We have developed an efficient and compact 3.4 μm difference-frequency-generation spectrometer using a 1.55 μm distributed feedback (DFB) laser diode, a 1.06 μm DFB laser diode, and a ridge-waveguide periodically poled lithium niobate. It is continuously tunable in the 30 cm^−1^ span and is applied to ^12^CH_3_D/^12^CH_4_ isotope ratio measurements. The suitable pair of ^12^CH_3_D ν_4_ ^p^P(7,6) and ^12^CH_4_ ν _2_+ν_4_ R(6) F_1_^(1)^ lines enabled us to determine their isotope ratio with a precision repeatability of 0.8‰ using a sample and a working standard of pure methane with an effective signal averaging time of 100 ms.

## 1. Introduction

Accurate isotope ratio measurements of environmental substances have been utilized to determine their production, transfer, and consumption processes. Conventionally, the isotope ratio has been determined by mass-spectrometer measurements because of the high sensitivity and accuracy possible with this technique. Recently, laser spectrometers have been applied to isotope ratio measurement, despite their relatively low sensitivity, because laser spectroscopy is more sensitive than mass spectrometry in some cases [1]. For example, it is able to distinguish isotopic molecules (isotopomers) with the same or almost equal weight (e.g., distinguishing ^15^N^14^N^16^O from ^14^N^15^N^16^O, and ^12^CH_3_D from ^13^CH_4_) without the dissociations or conversion processes that are usually required for mass spectrometry in such cases.

In the mid-infrared region, most molecules have strong absorption lines in the fundamental vibration band. However, light sources for mid-infrared spectroscopy have developed less remarkably than those in the near-infrared and visible regions. Recent development of tunable mid-infrared light sources, such as quantum cascade lasers and difference-frequency-generation (DFG) systems, has gradually driven their application to accurate spectroscopic measurement of trace gas, including isotope ratio measurements [1–11].

We have developed an efficient and compact 3.4 μm DFG light source using a 1.55 μm distributed feedback (DFB) laser diode, a 1.06 μm DFB laser diode, and a ridge-waveguide periodically poled lithium niobate (PPLN) [12]. The wavelength conversion of the PPLN is efficient, typically from 1 to 15%/W, which has been applied to molecular spectroscopy [13–16]. The particular device we used has an efficiency of 5 %/W; thus, pump and signal waves, even from low-power diode lasers, can be converted to 7.5 μW mid-infrared waves sufficient for linear absorption spectroscopy. The two DFB lasers provide a 30 cm^−1^ continuous tuning range, similar to reference [14], and optical fibers and fiber couplers reduce the labor involved in optical alignment. They also contribute to the compact and flexible design of the spectrometer.

The laser technique has been employed in isotope ratio measurement of methane [2,10,11,17–22] because it does not require the decompositions necessary in mass spectrometry. This is the initial report on such an application of the present spectrometer. Since it has a wider continuous tunable range in the strong fundamental band than those used in the previous works, we are able to choose an isotope pair appropriate for the measurement. Using pure sample gas, we have demonstrated that the spectrometer has potential for accurate isotope ratio measurements of environmental sample gas. To this end, it will be necessary to use an enhanced-cavity or multi-pass absorption cell and a preconcentration process because the ^12^CH_3_D/^12^CH_4_ ratio is 25 times smaller than the ^13^CH_4_/^12^CH_4_ ratio.

## 2. Methods

### 2.1. Experimental Setup

**Figure 1.**
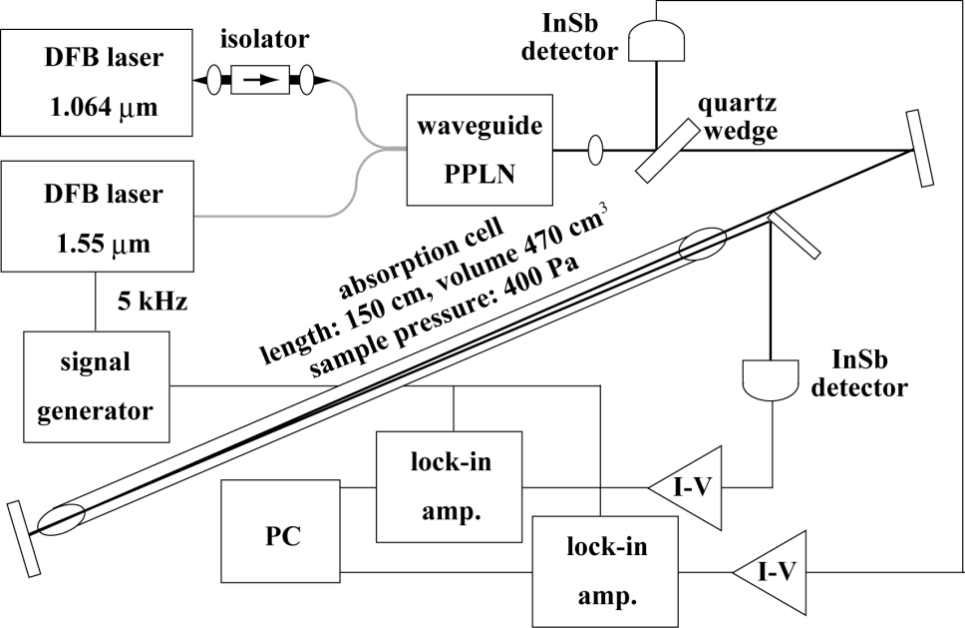
Schematic diagram of the experiment set-up. DFB: distributed feedback laser diode. I-V: current-voltage converter.

Figure 1 depicts a schematic diagram of the spectrometer. The DFG source consists of a ridge-waveguide PPLN (NEL, model WD-3360-000-A-B-C) with a conversion efficiency of 5 %/W, a 1.55 μm distributed feedback (DFB) laser diode module (Anritsu, model AB5A1102M521D) with a pigtail fiber output as a signal source, and a 1.064 μm DFB laser diode (Hamamatsu Photonics, model LA0927LP) as a pump source. The pump and signal lasers are each connected to an injection-current-temperature controller (ILX Lightwave, model LDC-3744B). The pump laser is mounted on a laser mount (Thorlabs, model LDM21) with a Peltier element, a thermistor, and a collimation lens.

The pump wave passes through an optical isolator (OFR, model IO-2.5-1064-VLP) and then an anamorphic prism pair (Thorlabs, model PS879-C), which transforms the beam shape from elliptical to circular for efficient coupling with an optical fiber. Half- and quarter-wave plates adjust the polarization of the pump wave for efficient wavelength conversion. The pump wave is then coupled with a polarization-maintaining (PM) single-mode fiber by a spatial-beam fiber coupler. The parallel-polarized signal (5 mW) and pump (30 mW) waves are combined by a PM fiber coupler and fed into the PPLN module through a pigtail fiber. A Peltier element and a thermistor in the PPLN module are connected with a temperature controller (ILX Lightwave, model LDT-5412) for phase matching. The generated 3.4 μm idler wave of 7.5 μW is sufficient for linear absorption spectroscopy, even though the total wavelength conversion efficiency is reduced due to loss at the spatial-beam fiber coupler, the PM fiber coupler, and three fiber connectors. The mid-infrared source is compact in dimension: the housings individually mounting the two DFB lasers and the PPLN are smaller than 100 cm^3^. The current sources and the temperature controllers used are typical commercially available products for low power consumption, and optical fibers reduce the number of mirror mounts and amount of space for optical alignment. The temperature tuning range of the individual laser device is 15 cm^−1^ without any frequency gap; therefore, the DFG source is tunable from 2,950 to 2,980 cm^−1^. The phase-matching condition of the PPLN is attained by temperature-tuning, and the single ridge-waveguide PPLN operates in the wavenumber range of 60 cm^−1^.

The idler wave is collimated by a sapphire lens and passes through a quartz wedge separating one tenth of the power level from the idler wave. The main beam enters a 150-cm-long absorption cell fitted with CaF_2_ windows, and the 470 cm^3^ volume is filled with 80 μmol of pure methane at a pressure of 400 Pa. The transmitted idler wave is sent back to the cell by a flat mirror, then reflected and focused onto a liquid-nitrogen-cooled InSb photodiode (Hamamatsu Photonics, model P5968-100) with the pump and signal waves removed by a 3.4 μm bandpass filter. The separated beam is immediately detected by another similar detector in order to monitor variations in the incident power level. The idler frequency is swept and modulated by superposing a 0.1 Hz triangle signal and a 5 kHz sinusoidal signal on the injection current of the 1.55 μm DFB laser. The modulation frequency is set at the highest response frequency of the detector [23,24]. The corresponding modulation depth is 250 MHz peak-to-peak, comparable with the absorption line width for obtaining a large signal without significant modulation broadening.

The detected signals are demodulated at 5 kHz (1*f* detection) by two lock-in amplifiers (Stanford Research Systems, model SR810) with a time constant of 10 ms. The 1*f* signals as well as the sweep signal are stored by the lock-in amplifiers at the acquisition rate of 500 Hz, corresponding to 2,500 data points in the up/down sweep of the idle frequency. The 1*f* signals are recorded ten times by the lock-in amplifiers, and the averaged spectrum is stored in a PC. The frequency sweep rate is limited by the time constant, the acquisition time, and the memory size of the lock-in amplifiers. The absorption cell is connected with a molecular turbo pump, an absolute pressure gauge (Edwards, 655AB) with an accuracy of 0.15 %, and two gas containers for exchanging sample and working standard gases. Neither the absorption cell temperature nor the room temperature is stabilized.

### 2.2. Absorption Lines for Isotope Ratio Measurement

Line selection has been discussed and analyzed [10,22,25,26]. Table 1 lists a pair of ^12^CH_4_ and ^12^CH_3_D lines with asterisks suitable for precisely measuring the isotope ratio together with the adjacent absorption lines having a line intensity exceeding 1.0 × 10^−25^ cm^−1^/(molecule·cm^−2^) [27]. The ν_4_ ^p^P(7, 6) line is one of the strongest lines of the less abundant ^12^CH_3_D. It does not seriously overlap the lines of ^12^CH_4_ and ^13^CH_4_, and it has a transition frequency and absorption intensity similar to those of the ν_2_ + ν_4_ R(6) F_1_^(1)^ line of ^12^CH_4_, which are favorable characteristics for measuring the isotope ratio. Therefore, the weak overtone band transition is chosen for the major isotope [10,22]. The absorption coefficient depends on temperature of −0.066 and −0.14 %/K at 295 K for the ^12^CH_3_D ν_4_ ^p^P(7,6) and ^12^CH_4_ ν_2_ + ν_4_ R(6) F_1_^(1)^ lines; therefore, the ratio has a low temperature dependence of 0.074 %/K, which is another advantage of the present pair.

**Figure 2.**
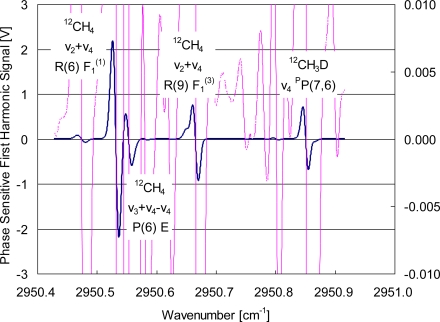
Recorded spectrum involving a suitable pair of ^12^CH_3_D and ^12^CH_4_ lines.

Figure 2 plots the observed spectrum involving these transitions. The absorption lines have a dispersion-like shape because of the 1*f* detection in wavelength-modulation spectroscopy. The thin red curve is drawn on a scale 300 times larger than the thick blue curve, and the vertical scale is indicated on the right side of Figure 2. Most small variations in the spectral curves come from the weak absorption lines partially listed in Table 1. The signal-to-noise ratio is limited by some periodic distortion with a typical magnitude of 0.001 V, which will be discussed in Section 3.2. The absorbance is 6 % for the ^12^CH_3_D ν_4_ ^p^P(7,6) line and 40 % for the ^12^CH_4_ ν_2_ + ν_4_ R(6) F_1_^(1)^ line at a sample pressure of 400 Pa. These values lie in the desirable range for sensitivity and signal linearity. The idler wave changes in output power as the injection current of the 1.55 μm DFB laser, and the power level at the ^12^CH_3_D ν_4_ ^p^P(7,6) line is 1.5 times higher than that at the ^12^CH_4_ ν_2_ + ν_4_ R(6) F_1_^(1)^ line. Therefore, residual amplitude modulation (RAM) inducing asymmetries in signal line shapes on wavelength modulation spectroscopy affects the 1*f* signal differently for the ^12^CH_3_D and ^12^CH_4_ lines. These asymmetries, however, do not affect the signal amplitude or detection sensitivity [24].

### 2.3. Isotope Ratio Determination

Here we define the signal ratio *R* as:

(1) $$R^{{\;}^{12}{\text{CH}}_{3}D}=I_{1f}^{{\;}^{12}{\text{CH}}_{3}D}/I_{1f}^{{\;}^{12}{\text{CH}}_{4}}$$

where *I*_1*f*_ is the peak-to-peak amplitude of the 1*f* signal. The values of *R* for the working standard (WS) and the sample are alternately measured. The *δ* value of the isotope ratio is then determined by:

(2) $$\delta^{12}{\text{CH}}_{3}D_{\text{Sample-WS}}=(R_{\text{Sample}}^{{\;}^{12}{\text{CH}}_{3}D}-R_{\text{WS}}^{{\;}^{12}{\text{CH}}_{3}D})/R_{\text{WS}}^{{\;}^{12}{\text{CH}}_{3}D}$$

Here, the value of *R*_WS_ is the average of the working-standard measurements immediately before and after the particular sample measurement to eliminate the effect of uniform signal drift discussed in Section 3.3.

The hydrogen isotope ratio is reported as a value relative to the international standard Vienna Standard Mean Ocean Water (VSMOW) for *δ*D. The value of *δ*^12^CH_3_D_Sample-WS_ is converted to that of *δ*^12^CH_3_D _Sample-VSMOW_ as follows:

(3) $$\delta^{12}{\text{CH}}_{3}D_{\text{Sample-VSMOW}}=\frac{1+\delta^{12}{\text{CH}}_{3}D_{\text{Sample-WS}}}{1+\delta^{12}{\text{CH}}_{3}D_{\text{WS-VSMOW}}}-1$$

### 2.4. Sample and Standard Gases

We measured the isotope ratio of the sample and the working standard of pure methane gas whose *δ*D_VSMOW_ was determined to be −289.9 ± 2.8 and −183.2 ± 1.5‰ using a gas chromatograph high-temperature conversion IRMS (GC/ TC/IRMS, Delta XL, Thermo Finnigan, Bremen, Germany).

The value of *δ*^12^CH_3_D (the isotopomer ratio) determined from the present laser measurements is easily converted to that of *δ*D-CH_4_ (the hydrogen isotope ratio of methane), because we use isotopically natural methane, for which the effects of other isotopomers (e.g., ^13^CH_3_D and ^12^CH_2_D_2_) are extremely small [28].

## 3. Results and Discussion

### 3.1. Sensitivity of the Spectrometer

To evaluate the spectrometer sensitivity, we recorded the 1*f* signal for an evacuated cell. We found a periodic distortion in the baseline with a period of 0.05 cm^−1^. This interference fringe is caused by weak reflection at the anti-reflection-coated interfaces of the 5-cm-long PPLN with a refractive index of 2.1. We reduced the distortion by dividing the mid-infrared beam into two, using a quartz wedge, and subtracting the 1*f* signal without any sample from that with the sample. In this way, spectra such as Figure 2 were recorded, attaining a detectable absorbance of < 1 × 10^−4^ with an effective signal averaging time of 100 ms. Accordingly, the signal-to-noise ratio was enhanced from 100 to 1,000 for the ^12^CH_3_D ν_4_ ^p^P(7,6) line. The resultant signal still had another smaller periodic distortion in the baseline with a period of 0.2 cm^−1^, probably because of the Brewster windows of the absorption cell. Distortion amplitude was sensitive to optical alignment and wavelength, limiting the minimum detectable coefficient to 4 × 10^−9^ cm^−1^·Hz^−1/2^. The sensitivity satisfied the necessary condition for determining the isotope ratio at an uncertainty level of 1 ‰.

### 3.2. Pressure Dependence of the Signal Ratio

**Figure 3.**
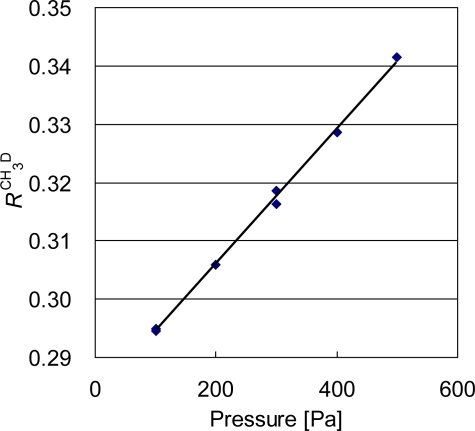
Pressure dependence of the signal ratio, R^CH_3_D^ = I_1f_^CH_3_D^/I_1f_^CH_4_^.

Figure 3 illustrates the pressure dependence of the signal ratio *R*^CH_3_D^ of Equation (1). The sample pressure is measured with the pressure gauge to an accuracy of 2 Pa. The large pressure dependence of 0.4 ‰/Pa at a pressure of 300 Pa is due to the difference of 5 to 1 in absorbance of ^12^CH_4_ to ^12^CH_3_D, and the exponential relation in the Lambert Beer's law between the absorbance and the pressure. Therefore, the pressure of the sample and the working standard must be identical with an uncertainty of 2.5 Pa for a 1-‰-level measurement.

### 3.3. Precision and Accuracy of the Isotope Ratio Measurement

**Figure 4.**
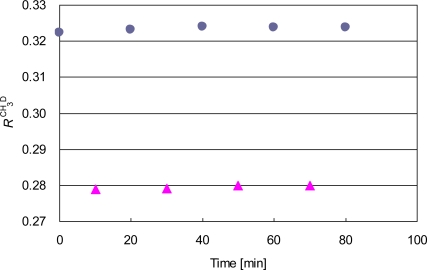
Alternate measurements of the signal ratio of ^12^CH_3_D/^12^CH_4_ of the working standard (circles) and the sample methane (triangles).

The absorption cell is alternately refilled with the working standard and the sample methane. The gas pressure is carefully set at (400 ± 1) Pa, and the value remains constant within ±1 Pa from the initial value during the measurement. Figure 4 depicts the temporal variation of the alternate isotope ratio measurements.

The plots of the sample and the working standard similarly drift by a relative magnitude of 0.46 and 0.36 % on a time scale of an hour, probably because of the temperature variation. If it were due to only the temperature dependence of the absorption coefficient ratio discussed in Section 2.2, the room temperature would change by 6 K. Since it is one order of magnitude larger than the actual condition, we attribute it to the other slow variations of the optical alignment and the electric circuits. To remove the slow effect, we use the averaging procedure described in Section 2.3. The values of *δ*^12^CH_3_D _Sample-VSMOW_ are then determined to be −294.2, −295.6, −293.7, and −293.5‰, yielding an average of −294.2‰ and the standard deviation of 0.8‰. The averaged *δ*^12^CH_3_D_Sample-VSMOW_ value is a little lower than the value of −289.9 ± 2.8‰ determined by a mass spectrometer. The difference is slightly beyond experiment uncertainty. This is the first report of the isotope ratio measurements by the present spectrometer. The result is tentative, and further measurements are required for comparison between laser spectroscopy and mass spectrometry.

## 4. Conclusions

We have developed a compact 3.4 μm DFG spectrometer using 1.06 and 1.55 μm DFB lasers and a waveguide PPLN, and precisely measured the ^12^CH_3_D/^12^CH_4_ ratio of pure methane having natural isotope abundance (^12^CH_3_D / ^12^CH_4_ < 0.1%). We determined the value of *δ*_VSMOW_ of the sample from four repetitive measurements using the working standard. The standard deviation is as small as 0.8‰, and the average is −294.2‰, which is slightly lower than −289.9 ± 2.8‰ determined by mass spectrometry.

## Figures and Tables

**Table 1.** Absorption lines of ^12^CH_3_D and ^12^CH_4_ in the vicinity of the pair for isotope ratio measurement (HITRAN 2008 [27]).

**Assignment**	**Wavenumber [cm^−1^]**	**Line intensity @296 K [cm^−1^/(molecule·cm^−2^) ]**	**Lower-level energy [cm^−1^]**
^12^CH_4_ 2ν_2_ P(14) F_1_^(1)^	2950.4819	2.826 × 10^−24^	1095.6320
*^12^CH_4_ ν_2_ + ν_4_ R(6) F_1_^(1)^	2950.5318	1.354 × 10^−22^	219.9411
^12^CH_4_ν_3_ + ν_4_ − ν_4_ P(6) E	2950.5519	2.914 × 10^−23^	1521.2847
^12^CH_4_ν_2_ + ν_3_ − ν_2_ P(5) E	2950.5596	4.464 × 10^−24^	1692.8063
^12^CH_4_ν_2_ + ν_4_ R(6) E	2950.5775	8.566 × 10^−25^	219.9133
^12^CH_4_ν_3_ + ν_4_ − ν_4_ P(11) F_2_^(1)^	2950.6175	5.102 × 10^−25^	1935.4170
^12^CH_4_ν_3_ + ν_4_ − ν_4_ P(9) A_2_^(2)^	2950.6428	1.415 × 10^−24^	1773.7814
^12^CH_3_D ν_4_^p^P (8,2)	2950.6489	6.712 × 10^−24^	284.5492
^12^CH_4_ν_2_ + ν_4_ R(9) F_1_^(3)^	2950.6601	4.011 × 10^−23^	470.8548
^12^CH_3_D 2ν_5_^r^Q (9,1)	2950.7784	1.872 × 10^−25^	350.1516
^12^CH_4_ν_2_ + ν_4_ R(9) F_1_^(1)^	2950.7982	5.009 × 10^−25^	470.7167
^12^CH_4_ν_3_ + ν_4_ − ν_4_ P(12) A_2_^(1)^	2950.8021	2.272 × 10^−25^	2101.1899
*^12^CH_3_D ν_4_^P^P (7,6)	2950.8508	2.734 × 10^−23^	266.3169
^12^CH_4_ν_3_ + ν_4_ − ν_4_ P(9) F_2_^(5)^	2950.8548	2.280 × 10^−25^	1775.9617
^12^CH_4_ν_1_ + ν_4_ − ν_4_ R(7) F_2_^(3)^	2950.8627	1.447 × 10^−24^	1599.2841
